# Lost in translation? Bridging the preclinical / clinical divide

**DOI:** 10.1186/1475-2875-11-S1-P112

**Published:** 2012-10-15

**Authors:** Mark Baker, Jörg Möhrle, Daren Austin

**Affiliations:** 1 Translational Medicine, Medicines for Malaria Venture, 1215 Geneva 15, Switzerland; 2 Quantitative Sciences, GlaxoSmithKline, Stockley Park, UK

## 

Establishing the relevance of preclinical data to the clinical situation is a problem that has plagued drug development in the majority of therapeutic areas. Typically a drug development cascade relies on preclinical data assuming the *in vitro* and *in* vivo data are consistent and predict some elements of clinical efficacy. Quantitative translation from preclinical to clinical is model based and requires PK (pharmacokinetics) and PK/PD (pharmacokinetic/pharmacodynamics) models linking drug exposure with effect.

The first modeling step undertaken for anti-malaria drug development at MMV has been to develop a model capable of linking preclinical *in vitro* to preclinical *in vivo* data to:

• Derive efficacy parameters for scaling including additional parameters that put them in context (parasite growth rates and maximum rates of killing)

• Determine the relationship and consistency between the *in vitro* and preclinical *in vivo* data.

This approach, whilst using data from a broader range of compounds and mechanisms, does assume that preclinical data is relevant replicating, at least partially, the clinical disease.

The *in vitro* data characterized the effects of fixed concentrations of compound on the reduction of *P falciparum* parasite concentrations and estimated IC50s. The *in vivo* assessment in SCID mice determined the compound’s dose-dependent reduction of *P falciparum* parasite concentrations and estimated an ID90. The *in vivo* data was modelled using a nonlinear mixed effects PK/PD model in Nonmem and its parameter estimates compared to those from the *in vitro* data.

The modeling was performed in a step-wise approach where a PK model was fitted to concentration time course data from a single dose in order to simulate the time courses used in the efficacy study (4 doses, 24 hours apart). The PK/PD model was then fitted to the observed parasitemia data using the simulated concentrations. The PD model estimated baseline parasitemia, rate constants for parasite growth and death, and the concentration dependent moderation of parasite death. The model determined that the parasite concentration expanded by approximately 3-4 times every 48 hours (in contrast to approximately 10 times in humans). For the two compounds modeled to date, model-estimated IC50 values from the *in vivo* data matched the *in vitro* estimates of IC50. The model, using the estimated parameters was also able to accurately simulate the decline in parasitemia and subsequent recrudescence following a single dose in the SCID *P falciparum* model providing some mechanistic validation.

This modeling is at an early phase requiring more data, preclinical and clinical, for validation including evaluating methods for the prediction of clinical PK. The agreement between *in vivo* and *in vitro* parameter estimates is encouraging and will be confirmed as Agreement between *in vitro* and *in vivo* parameters is encouraging as is the model’s ability to capture parasitemia dynamics. A simple approximation of this model is being evaluated for use in assessing MMV’s preclinical candidates. In addition to confirming the model’s preclinical utility, clinical data is being sought to test whether the model is equally able in predicting drug effects on *P falciparum* in humans, confirming the relevance of preclinical data.

